# Collagen-Polyvinyl Alcohol-Indomethacin Biohybrid Matrices as Wound Dressings

**DOI:** 10.3390/pharmaceutics10040224

**Published:** 2018-11-09

**Authors:** Ștefania Marin, Mădălina Georgiana Albu Kaya, Mihaela Violeta Ghica, Cristina Dinu-Pîrvu, Lăcrămioara Popa, Denisa Ioana Udeanu, Geanina Mihai, Marius Enachescu

**Affiliations:** 1 Department of Collagen, Division Leather and Footwear Research Institute, National Research and Development Institute for Textile and Leather, 031215 Bucharest, Romania; marinstefania.92@gmail.com (Ș.M.); madalina.albu@icpi.ro (M.G.A.K.); 2 Center of Surface Science and Nanotechnology, University Politehnica of Bucharest, 060042 Splaiul Independentei 313, Romania; geanina.mihai@cssnt-upb.ro (G.M.); marius.enachescu@cssnt-upb.ro (M.E.); 3 Department of Physical and Colloidal Chemistry, Faculty of Pharmacy, University of Medicine and Pharmacy “Carol Davila”, 20956 Bucharest, Romania; cristina.dinu@umfcd.ro (C.D.-P.); lacramioara.popa@umfcd.ro (L.P.); 4 Department of Clinical Laboratory and Food Safety, Faculty of Pharmacy, University of Medicine and Pharmacy “Carol Davila”, 020956 Bucharest, Romania; denisaudeanu@gmail.com; 5 Academy of Romanian Scientists, 010071 Bucharest, Romania

**Keywords:** collagen, indomethacin, wound dressing

## Abstract

The aim of this study is to design, develop and evaluate new biohybrid sponges based on polymers (collagen and polyvinyl alcohol) with and without indomethacin as anti-inflammatory drug model to be used for tissue regeneration in wound healing. Type I fibrillar collagen in the form of a gel and different concentrations of polyvinyl alcohol were mixed together to prepare composite gels. Both control samples, without indomethacin and with indomethacin, were obtained. All samples were crosslinked with glutaraldehyde. By freeze-drying of hydrogels, the spongious forms (matrices) were obtained. The matrices were characterized by FT-IR spectroscopy, scanning electron microscopy (SEM), water absorption, enzymatic degradation and in vitro indomethacin release. The pharmacological effect of the spongious biohybrid matrices was determined on an experimental model of burns induced to Wistar rats. The SEM images showed a porous structure with interconnected pores. Collagen sponges present a structure with pore sizes between 20 and 200 µm, which became more and more compact with polyvinyl alcohol addition. The FT-IR showed interactions between collagen and polyvinyl alcohol. The enzymatic degradation indicated that the most stable matrix is the one with the ratio 75:25 of collagen:polyvinyl alcohol (ACI75), the other ones being degradable in time. The kinetic data of indomethacin release from matrices were fitted with different kinetic models and highlighted a biphasic release of the drug. Such kinetic profiles are targeted in skin wound healing for which important aspects are impaired inflammation and local pain. The treatment with sponges associated with anti-inflammatory drug had beneficial effects on the healing process in experimentally induced burns compared to the corresponding matrices without indomethacin and the classical treated control group.

## 1. Introduction

Burns are the fifth most common cause of non-fatal injuries on children, according to World Health Organization [1]. Burns due to hot liquids or steam are more widespread than thermal burns and are more common for children [2]. Patients with severe burns are hospitalized in specialized departments and treated by plastic surgeons by temporarily covering wounds with allografts—transplanted tissue is obtained from a member of the same species, xenografts—transplanted tissue taken from a donor of one species and grafted into a recipient of another species—or dermal substitutes. Because allografts are expensive and difficult to obtain, xenografts are preferred, but they also require special treatments [3]. So, the best solution for treating burns remains the wound dressings. There are many options for wound dressings, but their selection must be based on factors such as the severity of the burn, the location of the wound, the water retention and the drainage, the frequency of dressing changing and, last but not least, the costs. The materials used to obtain dressings have to fulfill some essential conditions for an as fast as possible healing:

- Maintaining local moisture;
- Protecting the wound against infections;
- Absorbing the fluids released by the wound and exudates;
- Minimizing the uncovered surface;
- Preventing drying of the wound;
- Stimulating cell growth;
- To be elastic, non-toxic, non-allergenic, biocompatible and biodegradable [4].

Wound dressings can be obtained from natural or synthetic polymers. Thus, dressings obtained from natural polymers may exhibit adverse mechanical properties or cause immunological response, but also offer many advantages such as biocompatibility, biodegradability and the ability to be recognized by the body, which supports the cellular activity. Dressings produced from synthetic polymers do not have superior bioactive properties but have a well-defined structure that can be functionalized [5].

Type I collagen is the main protein component of mammalian connective tissue. It can be obtained and processed in various forms such as gels, sponges, films, fibers and hydrogels. All these forms of collagen exhibit high biocompatibility and biodegradability. Collagen is the most widespread protein in the human body having the ability to take on the function and shape of biological tissues. Also, due to its unique molecular organization, collagen has been used in many applications such as vascular grafting, fibrous materials for stem cell differentiation and matrices for regeneration of various types tissues [6]. It is the protein used predominantly to obtain biomaterials with different applications such as dressings, scaffold devices for tissue engineering, controlled release systems, composite materials for orthopedic field, etc. [7].

Polyvinyl alcohol is one of the most commonly used polymers in biomedical engineering and in the pharmaceutical industry due to its biocompatibility, its ability to crosslink structures without incorporation of toxic additives, and its simple structure, that can be easily adapted to the purpose of the applications [8]. Polyvinyl alcohol has been proven after many studies to be suitable for various biomedical applications including the basis for composite biomaterials because it is an excellent, non-toxic synthetic macromolecule [9]. It can be easily modified to acquire biological properties and is also suitable for making composite materials, especially with biological polymers such as chitosan or collagen [10].

Skin is the largest organ of the human body and has the role of protecting the internal tissues and cells against infections. Wounds from various sources are a real opportunity for microorganisms to invade and cause infections which slows down the healing process. Inflammatory agents increase vascular permeability, resulting in fibrin matrices generation, cause exudate and redness formation. In such cases an oral anti-inflammatory drug may be administered. The best option is, however, the development of bioactive matrices that can be used locally for wound healing. These pharmaceutical forms contain active care products that will facilitate and stimulate the healing process, inhibit infections by eliminating pathogens and releasing antibiotics [11].

Indomethacin is a non-steroidal anti-inflammatory drug. It is used to relieve pain and inflammation in rheumatic diseases, sprains, back pain, gout or menstrual pain. It acts by blocking in the body an enzyme called cyclooxygenase that is involved in the production of irritating chemicals in response to surgery or illness. By blocking the action of this enzyme, indomethacin is able to reduce the pain and inflammation [12].

Over the time, many biohybrids based on collagen and polyvinyl alcohol were developed. In 2018, Wang et al. developed a hydrogel with fish-originated collagen and polyvinyl alcohol, in which self-assembly of collagen and self-crosslinking of PVA were achieved. The study indicated that the prepared hydrogels presented ideal properties for tissue engineering. In 2015, Hameed et al. [10] examined matrices made of collagen, polyvinyl alcohol and bone particles, which are a mineral–collagen composite and demineralized bone, which gives naturally cross-linked collagen particles. Cell adhesion to the membranes was observed associated with the collagen particles indicating a lack of cytotoxicity, being excellent for tissue engineering use. Li et al. in 2018 [13] investigated the effect of nanofibers based on PVA/Collagen on epithelial–mesenchymal transition. This result indicated that 170 nm PVA/Collagen nanofibers induce A549 cells to process epithelial–mesenchymal transition more seriously than others nanofibers. Considering all the researches performed on biohybrids matrices we choose to develop a matrix made of collagen, polyvinyl alcohol and indomethacin to be used for wound healing taking in consideration the inflammatory process that may appear in case of injures.

Over the time, wound dressings based on collagen and polyvinyl alcohol have been developed by our team. In 2016 [14] we developed hydrogels based on collagen, polyvinyl alcohol and indomethacin which have been tested in terms of rheological analysis and antimicrobial activity. Based on the hydrogels performance, we concluded that the anti-inflammatory spongious matrices based on collagen and polyvinyl alcohol were potentially usable for burn injuries and wound healing. In 2017 [15] we tested those hydrogels in terms of enzymatic degradation in different pH and temperature conditions to demonstrate their ability to regenerate different types of wounds. The results showed that the hydrogels have the desired behavior because the wound pH, basic or acid, indicates a bad wound stage and the degradation should be faster in order to achieve faster regeneration. Considering the previous research in which wound dressings based on collagen and polyvinyl alcohol have been developed and characterized, the aim of this study was to develop a wound dressing for burns treatment consisting in collagen and polyvinyl alcohol with an anti-inflammatory drug, namely indomethacin, and also to characterize the matrices in terms of morphological properties, water absorption, enzymatic degradation, in vitro drug release and in vivo pharmacological studies.

In order to cover the complex pathophysiological process of the wound healing, several animal models were developed for a better target of the treatment. The most used models are the experimentally induced burns in Wistar rats, having some advantages due to similarities with human burns trauma, cellular and molecular interactions which are not able to be replaced by in vitro tests and low cost.

## 2. Materials and Methods

### 2.1. Preparation

The collagen and polyvinyl alcohol-based matrices were prepared using collagen gel, extracted according to the technology developed and used in the Collagen Research Department [16,17]. Polyvinyl alcohol (PVA) with a molecular weight of 60,000 was purchased from Sigma-Aldrich (Munich, Germany). Type I collagen (COLL) gel was extracted from bovine skin and the physical-chemical analysis resulted in concentration of about 2.85%. Collagenase used for enzymatic degradation studies was purchased from Sigma-Aldrich. Sodium hydroxide from Merck (Darmstadt, Germany) was of analytical grade and the water was distilled.

This gel with acid pH was adjusted to a concentration of 1% (*w*/*v*) and pH 7.2–7.4 (physiological pH) with 0.4 M NaOH solution. Subsequently, PVA was dispersed in the collagen gel in varying proportions and indomethacin (IND) as anti-inflammatory agent was added according to the compositions shown in Table 1. For crosslinking, 0.025% glutaraldehyde (GA) solution, purchased from Merck, was used.

10 different samples were obtained, namely C for collagen control sample, A for polyvinyl alcohol control sample, AC50 consisting in 50% collagen and 50% polyvinyl alcohol, AC75 consisting in 75% collagen and 25% polyvinyl alcohol and AC25 with 25% collagen and 75% polyvinyl alcohol. All samples containing indomethacin were coded with “I” such as CI (collagen–indomethacin), AI (polyvinyl alcohol–indomethacin).

**Figure 1:**
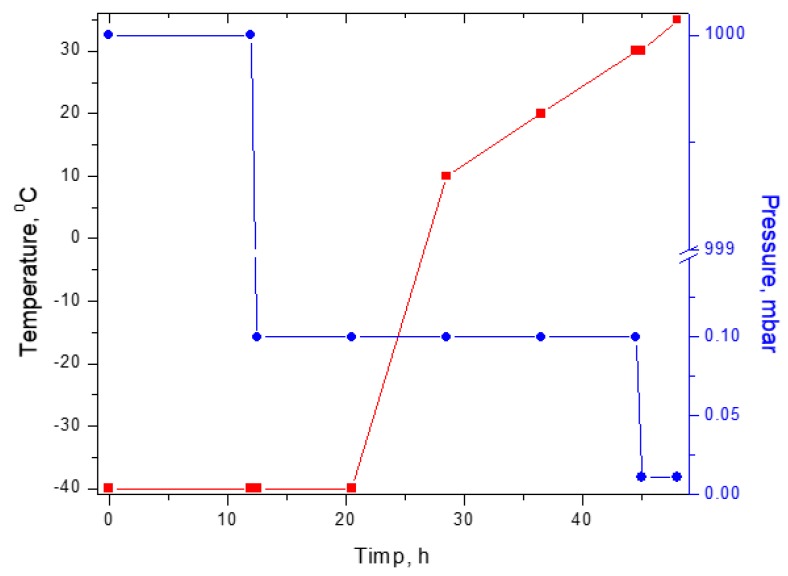
Lyophilization program used to obtain collagen–polyvinyl alcohol–indomethacin matrices.

After obtaining all the formulations, the gels were lyophilized using a Delta 2-24 LSC Christ lyophilizer, with a 48 h lyophilization program presented in Figure 1.

### 2.2. Water Absorption

To test the water absorption capacities, pieces of lyophilized samples (dried form) were initially weighed, immersed in water at 36 °C and then, at well-established time intervals, weighed again. The equation used (Equation (1)) to determine the absorption capacity was:

Water absorption [%] = *W_t_* − *W_d_*/*W_d_* (g/g), (1)

where, *W_t_* represents the weight of samples immersed at time *t*, and *W_d_* represents the weight of the dry sample. All samples were analyzed in triplicate for better accuracy of results [18].

### 2.3. Enzymatic Degradation

Enzymatic degradation of matrices was accomplished by immersing pieces of the hydrated lyophilized samples in a collagenase solution and monitoring the degradation over time. To monitor the weight loss, samples were weighed at well-defined time intervals, being extracted from the collagenase solution. The following formula is used:

Weight loss [%] = *W_i_* − *W_t_*/*W_t_* × 100 (2)

where *W_i_* represents the initial weight and *W_t_* represents the weight of the sample after the time interval *t* [15].

### 2.4. SEM (Scanning Electron Microscopy)

Scanning electron microscopy images were obtained using the equipment present within our group, namely a Hitachi SU 8230 Scanning Electron Microscope (Tokyo, Japan). Microstructural characterization was performed using the scanning electron microscope and all the lyophilized samples were analyzed in section without being coated with a conductive layer, taking advantage of low voltages imaging capabilities and/or deceleration voltages of the e-beam.

### 2.5. FT-IR

Spectral analyses were performed using the Jasco FT/IR 4000 Spectrophotometer (Tokyo, Japan). All spectra were recorded using the following parameters: spectral range 4000–600 cm^−1^, resolution of 4 cm^−1^ with 30 acquisitions per sample.

### 2.6. In Vitro Drug Release Studies

In vitro release of indomethacin from the designed spongious matrices was assessed using a dissolution equipment in conjunction with paddle stirrers (Esadissolver, Überlingen, Germany), as previously reported [19]. In brief, the pre-weighed samples were fixed in a transdermal sandwich device and then immersed in apparatus dissolution vessels. The release medium was the phosphate buffer solution of pH 7.4. During the kinetic determinations the release medium was uninterruptedly stirred at 50 rpm and the temperature was maintained at 37 °C ± 0.5 °C. 5 mL samples from the dissolution vessel were withdrawn at different time intervals over a period of 8 h/24 h and replaced with an equal volume of fresh, pre-heated phosphate buffer solution to sustain the equilibrium constant. The concentration of indomethacin released in the medium was spectrophotometrically evaluated (Perkin-Elmer UV-Vis spectrophotometer, Milan, Italy) at an absorbance of 268 nm [20], using the previously determined linear calibration curve, *R*^2^ = 0.9990) [21]. The release studies were performed in triplicate.

The kinetic profiles recorded as cumulative released drug percentage versus time were built and the experimental data were fitted using the Power law kinetic model (Equation (3)) and its particular cases, Higuchi (*n* = 0.5) and Zero-Order (*n* = 1):

*m_t_*/*m*_∞_ = *k* ∗ *t^n^* (3)

where *m_t_*/*m*_∞_ is the fractional drug released at time *t*, *k*—the kinetic constant, *n*—the release exponent related to the drug transport mechanism [22,23].

### 2.7. Pharmacological Studies

#### 2.7.1. Animal Model Description of Experimentally Induced Burns

The 2–3 month old Wistar rats weighing 170 ± 10 g were provided by the Animal Biobase of the “Carol Davila” University of Medicine and Pharmacy, Bucharest, Romania.

All animals used in the study were kept in standard laboratory conditions, were fed twice a day and received water ad libitum. The experiment was performed in compliance with the European Communities Council Directive 2010/63/UE and Law No. 43 of the Romanian Parliament from 11 April 2014 (ethic approval number 2143 from 6 June 2018).

The animals were divided in 11 groups (*n* = 6) as follows: Group 1—C, Group 2—CI, Group 3—A, Group 4—AI, Group 5—AC25, Group 6—ACI25, Group 7—AC50, Group 8—ACI50, Group 9—AC75, Group 10—ACI75, Group 11—Control.

The dorsal area of each animal was shaved off to ensure a proper burn wound due to the difficulties for the experimental rats to reach the lesion and to induce further injuries. The animals were anesthetized with ether ethylic and the burn was induced using a special stainless-steel cylindrical device of 10 mm diameter previously heated at 100 °C in boiling physiological serum and applied on the shaved dorsal area for 15 s. According to groups 1–10, different collagen scaffolds were applied once in the first day of the study on the affected area and were fixed with a silk plaster. The control group used in this study was classically treated with sterile cotton dressing usually used for burns, the dressing being changed daily for the first three days [19,24,25]. The surface morphology of the wounds was recorded for 14 days using a digital camera (Olympus SP-590UZ) in the absence of scaffold. The wound diameter profile was measured in days 1, 3, 5, 7, 10, and 14. The wound was considered healed after the scar fell off.

The wound healing process was calculated according to the size profile of wound as described by the following equation (Equation (4)):

(4) $$\;Re-epithelization\;\%\;=\frac{\left(W_{ound\;size\;at\;t=0}\right)-\left(W_{ound\;size\;at\;t}\right)}{W_{ound\;size\;at\;t=0}}\;\times 100\;$$

where the wound size was an average measure of the longest and shortest dimensions of the affected area [19,26].

Any aspects of discomforts, pain and other modification on the animal health status as well as inflammation or infection of the wounds were also daily monitored.

#### 2.7.2. Statistical Analyses

Statistical analyses were performed using the GraphPad Prism 6 and Excel software. The experimental data were expressed as mean and standard deviations (SD) and were evaluated using the student *t*-test and analyses of variance followed by Dunnett’s multiple comparison test. The results were considered significant at *p* < 0.05, high significant at *p* < 0.01 and not significant at *p* > 0.05.

## 3. Discussion

**Figure 2:**
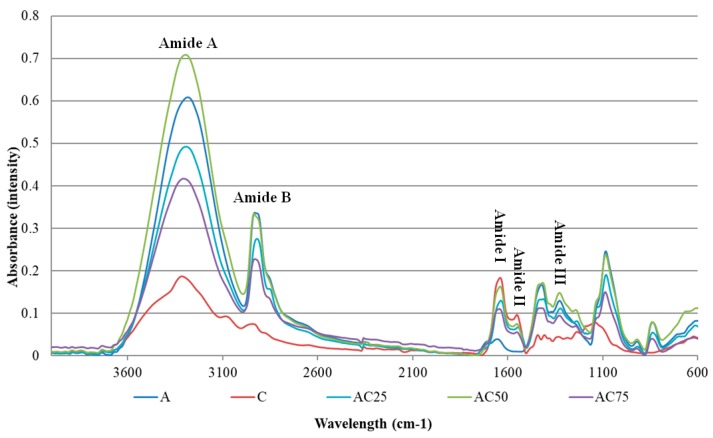
FT-IR spectra of obtained matrices for control samples.

In Figure 2 FT-IR spectra are presented for all the obtained matrices.

It can be observed from both spectra that polyvinyl alcohol show bands similar to those found in the literature [27], namely the absorbing behavior at 3281 cm^−1^ (O–H stretching vibration) due to inter and intramolecular hydrogen bonds. The vibration band from 2930 cm^−1^ refers to the C-H stretch of the alkyl groups, and the peak from 1745 cm^−1^ is due to the C=O and C–O stretches, from the remaining acetate groups in the PVA [28].

Regarding the sample C, the control sample of collagen, it can be seen in the above figure the specific peaks for collagen, amide A at 3312 cm^−1^, amide B at 2938 cm^−1^, amide I at 1638 cm^−1^, amide II at 1553 cm^−1^, amide III at 1245 cm^−1^, strips specific to the collagen structural organization.

For samples that contain both COLL and PVA, it can be seen that the bands for amide A and B of the COL are not altered, but collagen amide I, II and III are very difficult to be identified due to the very small absorbance. The samples retained the structure of polyvinyl alcohol and some peaks overlapped completely, especially in the sample AC25, which has the highest PVA amount. No changes were observed at the time of indomethacin addition, spectra being similar.

**Figure 3:**
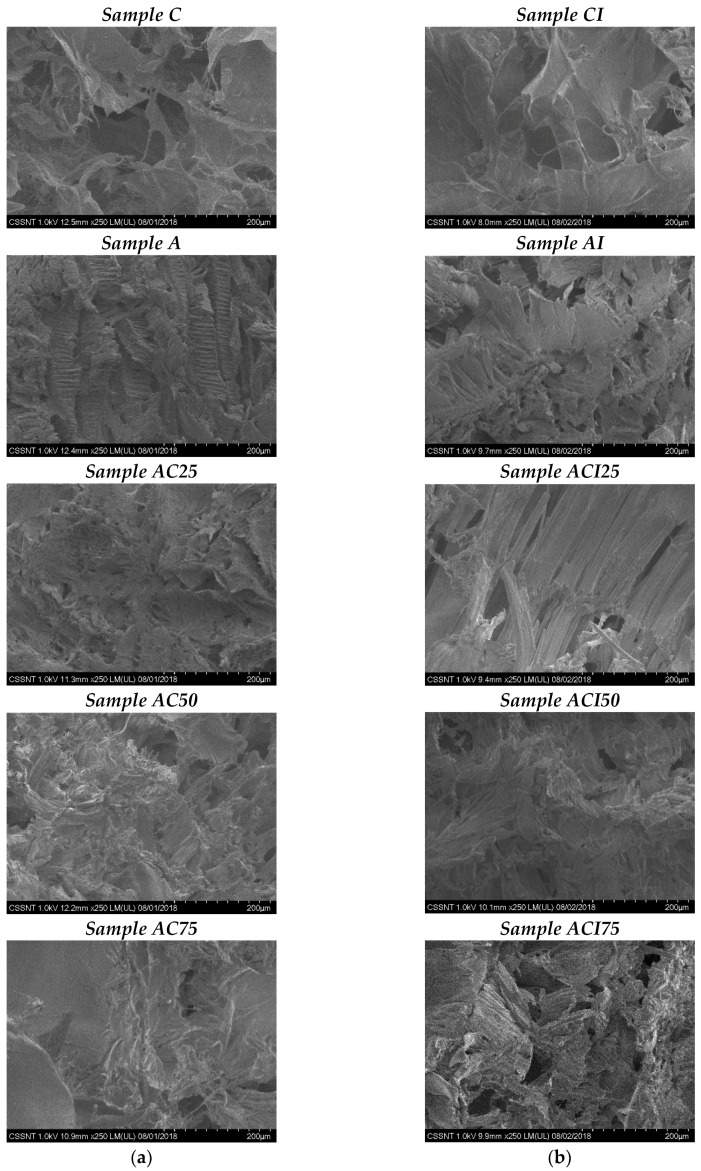
SEM images of (**a**) control samples, without indomethacin and (**b**) matrices with indomethacin, 250×.

The obtained images are presented in Figure 3.

First of all, the structure of the polyvinyl alcohol can be noticed. Samples A and AI successfully illustrate the specific structure of polyvinyl alcohol, namely lamellar formations, oriented, with a rigorously defined structure. Moreover, an increase in porosity is observed for all samples that contain indomethacin, due to the alteration of PVA structure. In comparison with the other samples, the presence of PVA, can be noticed in all samples, the specific lamellar formations being interspersed in the collagen-specific structure. The samples C and CI, have the collagen-specific structure, characterized by the interconnected pores shaped by the fibrils, pores with dimensions of approximately 20–200 μm.

In the case of samples AC50/ACI50 and AC75/ACI75, the homogeneous mixture between PVA and collagen structure can be observed. The high porosity is predominantly remarked for sample ACI75, with a larger amount of collagen, but also for ACI50. Regarding sample AC25/ACI25, the PVA structure is predominant, which is predictable considering the 75% amount of PVA. Also, the sample density is much higher compared to the ones with a larger amount of collagen. By the addition of indomethacin, no major structural changes were observed so the control samples have the same structure compared to the ones with indomethacin.

**Figure 4:**
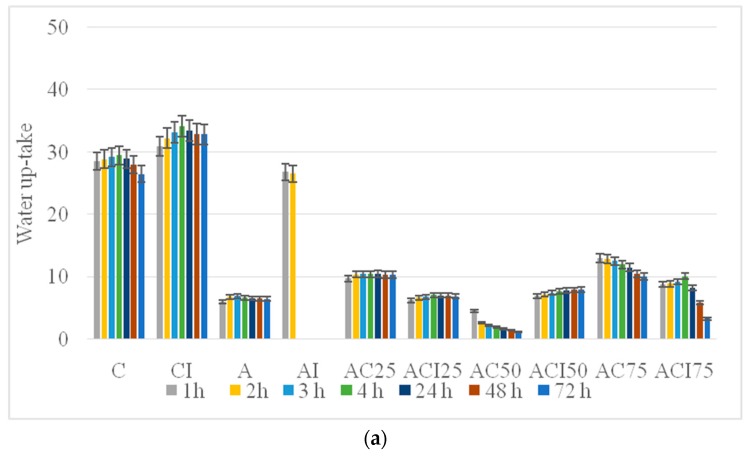

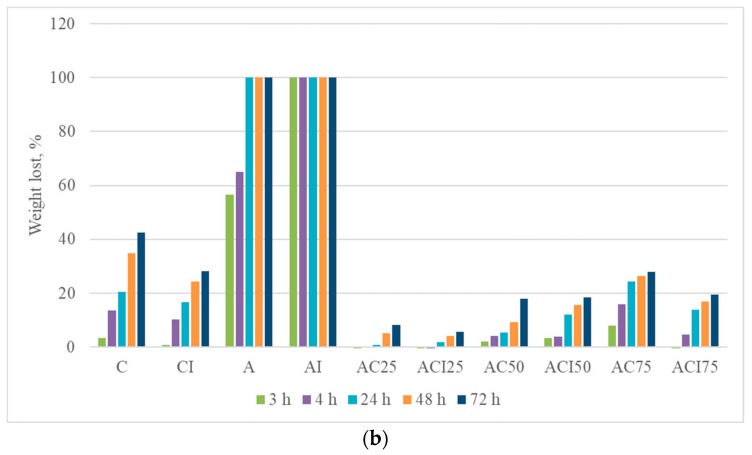
Water up-take (**a**) and enzymatic degradation (**b**) for all spongious matrices.

The absorption capacity is shown in Figure 4a and the enzymatic degradation in Figure 4b for all matrices. Tests were performed over 72 h and all the samples were analyzed in triplicate.

We can see in Figure 4a, firstly, that collagen sample C, without indomethacin and CI with indomethacin have the highest absorption capacity, absorbing about 30 g/g respectively 35 g/g. The polyvinyl alcohol control sample has the lowest absorption capacity of about 7 g/g. The same behavior is noticed for the sample with polyvinyl alcohol and indomethacin, this one having the absorption capacity of 3 g/g, in line with our expectations because the collagen matrices C and CI show high porosity and the water can be absorbed into the polymeric networks, while the PVA matrix A exhibits high density and low absorption capacity. Beside all of that, it can be also observed the influence of indomethacin: for collagen matrices the absorption capacity is higher for the sample that contains the anti-inflammatory agent while for PVA matrices the absorption is smaller for the one with indomethacin, who degraded after 1 h, but the degradation takes place faster which may mean porosity increase for both cases.

Samples AC50/ACI50, AC75/ACI75 and AC25/ACI25, for which the ratio of collagen: polyvinyl alcohol varied, have a significant absorption capacity of between 8 and 15 g/g, depending on the proportion of collagen and PVA. Collagen in higher proportions provides porous structures, while the predominant PVA amount presents more compact structures with less water absorption. Thus, we can see from Figure 4 that the AC75 sample having in composition 75% collagen has the highest absorption capacity, around 14 g/g, whereas the sample AC25 having only 25% collagen in composition has a capacity of absorption significantly lower, approximately 10 g/g, due to PVA density. For sample AC50 where the collagen-polyvinyl alcohol ratio is the same, we can see an initial absorption tendency of about 6 g/g in the first hour, followed by a gradual degradation over the next 72 h, due to collagen degradation from the sponge support. Therefore, the sample with the highest absorption capacity was AC75 consisting of 25% PVA and 75% COL.

A higher stability but a lower absorption capacity was noticed for samples with indomethacin. In this case the samples are not degraded as fast as the ones without anti-inflammatory agent but the absorption is lower namely: sample ACI50 has an absorption capacity of 8 g/g compared with AC50 with an absorption capacity of 7 g/g and the degradation process starts after 24 h; sample ACI75 has an absorption capacity of 10 g/g compared to 13 g/g absorption capacity of AC75 but the degradation in case of ACI75 starts after 4 h compared to 1 h for AC75. The sample with the lowest amount of collagen, ACI25, absorbs 7 g/g compared to its control sample AC25 which has an absorption capacity of 10 g/g but the degradation starts after 48 h and not after 24 h as it happened for AC25. Thus, it can be noticed that the porosity of the samples, respectively their absorption capacity, increases proportionally with the increase of the collagen percentage, taking into account that the concentration of the crosslinker was the same, results that can be correlated with the microscopic images.

The enzymatic degradation is shown in Figure 4b for all matrices. Tests were performed over 72 h and all samples were analyzed in triplicate by their dipping into collagenase solution and subjecting to a temperature of 37 °C, in order to simulate the physiological conditions.

As it can be seen, for control sample for polyvinyl alcohol—A and AI the degradation took place completely in the first hour of testing for AI and in 24 h for A. If we refer to control samples, those without indomethacin, the lowest degradation percentage is observed in case of the sample AC25, showing a degradation of 8%, and the higher degradation was recorded by sample AC75, 27%, which was expected taking in consideration that this sample contains more collagen than sample AC25, proving that the collagenase solution has the ability to cleave collagen fibers and, in the same time, the PVA gives the sample density. The same behavior was observed for hybrid samples with indomethacin: the degradation percentage was higher for samples with a higher amount of collagen. However, all hybrid matrices tested retain their integrity even after 72 h.

**Figure 5:**
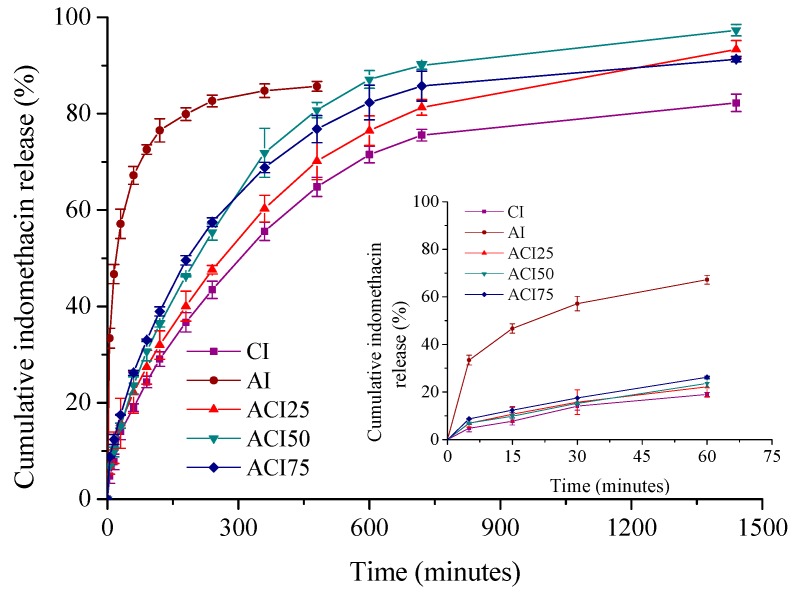
Time-dependent cumulative release profiles of indomethacin from spongious matrices.

The kinetic studies represent an important stage for the characterization of wound dressing loaded with an anti-inflammatory drug intended to control the inflammatory process and subsequently to alleviate the pain associated to a cutaneous lesion. The suitability of the designed formulations was monitored in terms of collagen: polyvinyl alcohol ratio influence on the indomethacin release by comparing the kinetic patterns. Thus, the time evolution of the in vitro drug cumulative release is presented in Figure 5.

All systems showed a typical biphasic kinetic profile with an initial burst release effect during the first hour, followed by a prolonged and controlled drug release for the next 7 h (formulation AI containing polyvinyl alcohol only), respectively 23 h (collagen supports with a variable polyvinyl alcohol content—CI, ACI25, ACI50 and ACI75).

For the formulation like polyvinyl alcohol-based spongious matrix (AI), the burst release effect is very marked in the first 60 min (~67%). In turn, for collagen spongious matrices with various PVA ratios, the burst release effect is less evident (Figure 5), the drug being released slower in the first 60 min, approximately between 19% (CI) and 26% (ACI75 with a 75:25 Col:PVA ratio).

The cumulative indomethacin released percentage after 8 h is of 85.68% for the sample containing polyvinyl alcohol only, while for the collagen supports having a variable polyvinyl alcohol content it varies after 24 h between 82.28% (sample containing collagen only—CI) and 97.34% (binary composite sponge with a ratio 50:50 collagen:polyvinyl alcohol—ACI50) (Table 2).

The results highlighted that both collagen support and new developed hybrid collagen/polyvinyl alcohol sponges have the capacity to retain the drug and deliver it in a controlled manner for a longer period of 24 h.

Such kinetic profiles are targeted when an immediate drug action is required to control the pain and the inflammation after injury, followed by a prolonged release during several hours, securing the anti-inflammatory and analgesic local effect for a longer period needed for wound healing, the first 12–48 h being decisive in wound healing process.

The drug release mechanism was investigated by fitting the in vitro experimental data with Peppas model (Equation (5)) and its particular cases, Higuchi (*n* = 0.5) and Zero-Order (*n* = 1), the corresponding correlation coefficients values being given in Table 1.

As noticed from Table 2, the highest values for the correlation coefficient were obtained for Power model, indicating that the drug release data were best fitted with the above kinetic model, the kinetic parameters characteristic to this model being shown in Table 2.

Thus, the release exponent values in the range 0.18–0.43 reflect a non-Fickian indomethacin transport mechanism from the designed supports, indicating that several physical-chemical processes are involved in the drug release, as previously reported by us and also by other authors [19,23,29,30]: (1) an initial desorption of the drug retained at the sponge surface; (2) absorption of the release medium by the hydrophilic biopolymeric sponges followed by their swelling; (3) progressive transformation of spongious matrix in gel; (4) diffusion of the drug immobilized in the collagen fibrillar/PVA structure concomitantly with (5) gradual erosion of the release support.

The healing process of burn injuries is very complex and comprises four main superimposing phases (hemostasis, inflammation, proliferation, and tissue remodeling) activated at a specific time with a proper intensity depending on different topic or systemic factors. The tissue homeostasis and post-traumatic inflammation processes are critical for the evolution of tissue repairing and remodeling process and represent the first target of a successful treatment of burns [31].

An increased level of pro-inflammatory cytokines is a consequence of the activation of the inflammation signaling pathways to stimulate neutrophils migration and macrophages activation.

Several other cell types like fibroblasts and keratinocytes with specific biological roles are activated during the repairing process with important activities in reconstructing the first defense barrier. Strong inflammatory signals secreted at the affected area interfere with the following healing stages by maintaining the environmental stress and promoting the formation of thick scars by increasing the secretion of extracellular matrix [31].

The therapeutic approach should consider the complex phases of the cicatrizing process and must target the most appropriate stage to obtain fast wound healing and minimal scaring.

Short term use of non-steroidal anti-inflammatory drugs like indomethacin applied in topical formulation proved to be efficient in reducing the local pain and generation of normal skin comparing to long term use of NSAIDs with impaired wound healing [19,32].

**Figure 6:**
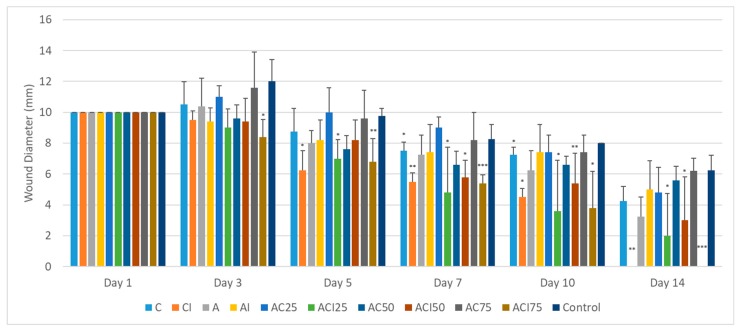
The variation of wound diameter (mm) in days 1, 3, 5, 7, 10 and 14 after inflicting the burns and the treatment with collagen scaffolds. The graph bars represent standard deviation. Dunett’s test (vs. control) * *p* < 0.05, ** *p* < 0.01, *** *p* < 0.001 (C = collagen, I = indomethacin, A = polyvinyl alcohol).

**Figure 7:**
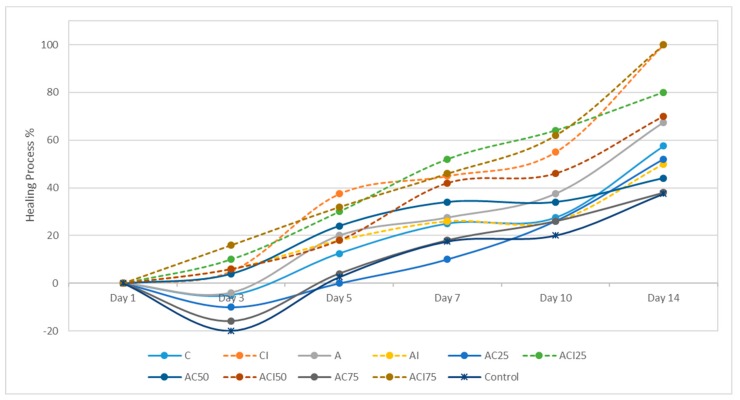
The evolution of the healing process for 14 days after inducing the burns and the treatment with spongious matrices (C = collagen, I = indomethacin, A = polyvinyl alcohol).

**Figure 8:**
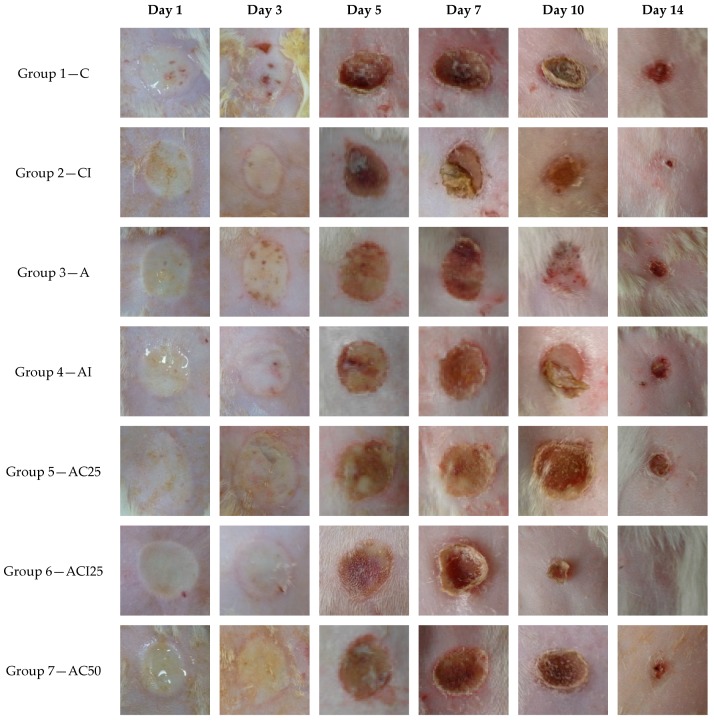

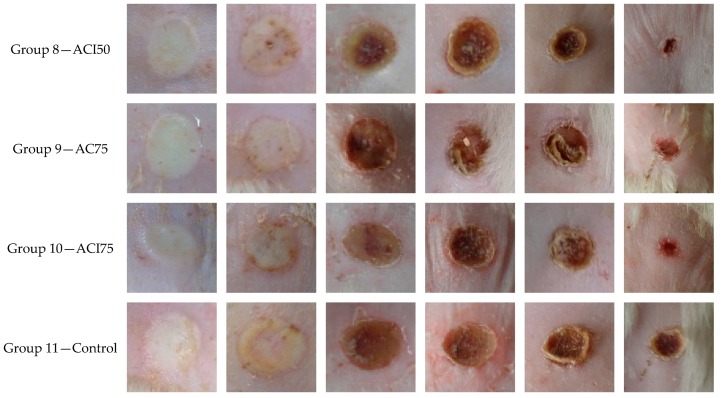
The macroscopic evolution of the wound morphology after different treatment with spongious matrices and control group for 14 days (magnification 1.2×).

The effect of the treatment with spongious matrices with different ratios of collagen: polyvinyl alcohol, and/or indomethacin on the cicatrizing process of experimentally induced burns on Wistar rats was monitored for 14 days and evaluated at different time intervals at 1, 3, 5, 7, 10, and 14 days (Figure 6, Figure 7 and Figure 8) in comparison with control group. The groups 2 (CI), 4 (AI), 6 (ACI25), 8 (ACI50) and 10 (ACI75) were treated with spongious matrices containing indomethacin and their pharmacological effect was compared with the effect of the same sponges without anti-inflammatory agent from groups 1 (C), 3 (A), 5 (AC25), 7 (AC50), and respectively 9 (AC75).

After inducing the experimental burn on rats, the affected area was characterized by a white eschar with erythematous edges. The skin layers, epidermis, and dermis were severely affected, and the wound soon become fully hyperemic due to the red blood cells extravasation and activation of post-traumatic inflammation process.

The spongious matrices were applied after inflicting the burns to minimize the local damage in the first critical stage of injury evolution by suppling a scaffold-like matrix to favor the migration of the cells involved in repairing the first defense barrier.

After 3 days from burn injury, a slightly post-traumatic inflammatory process was noticed in case of the following groups: control, group 9 and group 7 with a slow healing process (Figure 6 and Figure 7). The treatment with sponges loaded with indomethacin in groups CI, ACI25, ACI50, ACI75 decreased the wound diameter promoting the tissue repairing compared to the same sponges without anti-inflammatory agent. A significant cicatrizing process was registered for group ACI75 (*p* < 0.05) followed by group ACI25 with a 26% and respectively 10% decrease of wound diameter (Figure 7).

In case of group ACI75, the healing process was significantly faster after 10 days with almost complete re-epithelization and minimal scaring of the affected area in case of almost all animals of the group (Figure 8). Comparing the results obtained in case of the groups CI, ACI50 and ACI25, the healing rate was similar with no significant differences (*p* > 0.05) after 10 days from the experimental injury.

After 14 days, the healing was significantly advanced compared to control for groups 8 (ACI75), 2 (CI), 6 (ACI50), and 10 (ACI25). All animals of groups CI and ACI75 were healed (*p* < 0.01) after two weeks compared to control group with a delayed tissue repairing and thick scars.

All groups treated with spongious matrices presented a faster cicatrizing process compared to the classically treated control group. An evident beneficial effect of ACI75, CI, ACI25 and ACI50 sponges was explained by the presence in the formulation of collagen with a structure similar to the natural endogenic derivative polypeptides having an important biological role in maintaining and regenerating the skin barrier function. Moreover, in case of the spongious matrices loaded with a topical anti-inflammatory agent (indomethacin), a synergic pharmacological effect was observed on the long-term healing process of the experimental burns induced to Wistar rats.

## 4. Conclusions

SEM microscopy images show the morphology of lyophilized samples. Collagen shows a porous structure with pores between 20 and 200 μm, which becomes more compact when PVA is added; FT-IR spectra showed the interactions between collagen and polyvinyl alcohol; due to the combination of collagen and polyvinyl alcohol, the intensity of amides I, II, and III decreases, but they are at the same wavelengths, which means that collagen is not distorted during interactions, preserving its biological properties. Water up-take and in vitro enzymatic degradation studies showed that most of the samples are stable.

The kinetic profiles indicated that the new hybrid collagen/polyvinyl alcohol composite sponges show a moderate burst release effect of indomethacin during the first hour and present a controlled and sustained drug release for the subsequent 23 h. This release is promising both for reducing fast persistent pain and inflammation after wound occurrence and offering an anti-inflammatory and analgesic local effect over the longer period needed for burn healing.

The treatment with spongious biohybrid matrices demonstrated a beneficial effect on the healing process in case of experimental induced burns to Wistar rats in comparison with the control group. The cicatrizing process was faster in case of all groups treated with sponges associated with anti-inflammatory drug compared to the similar sponges without drug. The most promising pharmacological results were obtained in case of the treatment with ACI75 composite sponge, followed by CI, ACI25 and ACI50. Corelating all the results obtained from the pharmacological tests but also from the physical-chemical and biopharmaceutical analysis, the sample COLL:PVA = 75:25, ACI75 showed the most promising results so it could be selected for further in vivo studies.

The complex results of physical-chemical, biopharmaceutical, biological, and pharmacological evaluation of the designed spongious biohybrid matrices recommend these formulations as drug delivery systems with potential applications in the treatment of skin wounds.

## Figures and Tables

**Table 1:** Composition of collagen–polyvinyl alcohol matrices doped with indomethacin.

Sample Code	C	CI	A	AI	AC25	ACI25	AC50	ACI50	AC75	ACI75
Collagen (COLL), %	100	100	0	0	25	25	50	50	75	75
Polyvinyl alcohol (PVA), %	0	0	100	100	75	75	50	50	25	25
Indomethacin (IND), %	0	0.2	0	0.2	0	0.2	0	0.2	0	0.2
Glutaraldehyde (GA), %	0.025	0.025	0.025	0.025	0.025	0.025	0.025	0.025	0.025	0.025

**Table 2:** Correlation coefficients for indomethacin release from spongious supports determined by application of Power law, Higuchi and Zero-order models; kinetic parameters specific for Power law model; drug released percentage.

Matrices	Kinetic Constant (1/min)	Release Exponent	Power Model	Higuchi Model	Zero-Order Model	Drug Released (%)
AI	0.305	0.18	0.9876	0.8675	0.7017	85.68 *
CI	0.038	0.43	0.9811	0.9766	0.8727	82.28 **
ACI25	0.041	0.40	0.9883	0.9844	0.8893	93.35 **
ACI50	0.053	0.42	0.9761	0.9687	0.8538	97.34 **
ACI75	0.066	0.38	0.9788	0.9640	0.8373	91.31 **

* Drug released percentage after 8 h; ** drug released percentage after 24 h.
